# A collaborative, computer-assisted, psycho-educational intervention for depressed patients with chronic disease at primary care: protocol for a cluster randomized controlled trial

**DOI:** 10.1186/s12888-021-03380-2

**Published:** 2021-08-21

**Authors:** Graciela Rojas, Pablo Martínez, Viviana Guajardo, Solange Campos, Pablo Herrera, Paul A. Vöhringer, Víctor Gómez, Wilsa Szabo, Ricardo Araya

**Affiliations:** 1 Departamento de Psiquiatría y Salud Mental, Hospital Clínico Universidad de Chile, Avenida La Paz, 1003 Santiago, Chile; 2 ANID, Millennium Science Initiative Program, Millennium Institute for Depression and Personality Research (MIDAP), Santiago, Chile; 3 ANID, Millennium Science Initiative Program, Millennium Nucleus to Improve the Mental Health of Adolescents and Youths, Imhay, Santiago, Chile; 4 ANID, Millennium Science Initiative Program, Millennium Nucleus in Social Development (DESOC), Santiago, Chile; 5 Escuela de Psicología, Facultad de Humanidades, Universidad de Santiago de Chile, Santiago, Chile; 6 Psicomedica, Clinical & Research Group, Santiago, Chile; 7 Servicio de Psiquiatría, Hospital El Pino, Santiago, Chile; 8 Escuela de Enfermería, Facultad de Medicina, Pontificia Universidad Católica de Chile, Santiago, Chile; 9 Escuela de Psicología, Facultad de Ciencias Sociales, Universidad de Chile, Santiago, Chile; 10 Mood Disorders Program, Tufts Medical Center, Boston, MA USA; 11 Department of Psychiatry, Tufts University School of Medicine, Boston, MA USA; 12 Facultad de Medicina, Universidad de Chile, Santiago, Chile; 13 Programa de Doctorado en Psicoterapia, Facultad de Medicina y Facultad de Ciencias Sociales, Universidad de Chile y Pontificia Universidad Católica de Chile, Santiago, Chile; 14 Department of Health Services and Population Research, King’s College London, London, UK

**Keywords:** Study protocol, Cluster randomized trial, Depression, Hypertension, Diabetes, Primary care, Cognitive-behavioral, Psychotherapy

## Abstract

**Background:**

Depression and chronic diseases are frequently comorbid public health problems. However, clinical guidelines often fail to consider comorbidities. This study protocol describes a cluster randomized trial (CRT) aimed to compare the effectiveness of a collaborative, computer-assisted, psycho-educational intervention versus enhanced usual care (EUC) in the treatment of depressed patients with hypertension and/or diabetes in primary care clinics (PCC) in Santiago, Chile.

**Methods:**

Two-arm, single-blind, CRT carried out at two municipalities in Santiago, Chile. Eight PCC will be randomly assigned (1:1 ratio within each municipality, 4 PCC in each municipality) to the INTERVENTION or EUC. A total of 360 depressed patients, aged at least 18 years, with Patient Health Questionnaire-9 Item [PHQ-9] scores ≥15, and enrolled in the Cardiovascular Health Program at the participating PCC. Patients with alcohol/substance abuse; current treatment for depression, bipolar disorder, or psychosis; illiteracy; severe impairment; and resident in long-term care facilities, will be excluded. Patients in both arms will be invited to use the Web page of the project, which includes basic health education information. Patients in the INTERVENTION will receive eight sessions of a computer-assisted, psycho-educational intervention delivered by trained therapists, a structured telephone calls to monitor progress, and usual medical care for chronic diseases. Therapists will receive biweekly and monthly supervision by psychologist and psychiatrist, respectively. A monthly meeting will be held between the PCC team and a member of the research team to ensure continuity of care. Patients in EUC will receive depression treatment according to clinical guidelines and usual medical care for chronic diseases. Outcome assessments will be conducted at 3, 6, and 12 months after enrollment. The primary outcome will be depression improvement at 6 months, defined as ≥50% reduction in baseline PHQ-9 scores. Intention-to-treat analyses will be performed.

**Discussion:**

This study will be one of the first to provide evidence for the effectiveness of a collaborative, computer-assisted, psycho-educational intervention for depressed patients with chronic disease at primary care in a Latin American country.

**Trial registration:**

retrospectively registered in ClinicalTrials.gov, first posted: November 3, 2020, under identifier: NCT04613076.

## Background

### Background and rationale

According to the Global Health Estimates by the World Health Organization (WHO), non-communicable diseases, such as cardiovascular diseases and diabetes, accounted for 68.4% of total deaths worldwide in 2015 [1]. Up to date global burden of disease data also reveals that depressive disorders are the single largest cause of global disability, leading to considerable losses in health and functioning and contributing to nearly 800,000 deaths by suicide per year [2, 3]. Population ageing means that cardiovascular diseases and diabetes are becoming more prevalent, while depression might further limit the capacities of this ever increasing older workforce [3, 4]. Moreover, studies have revealed that depression comorbid with medical conditions is the rule rather than the exception, as up to two thirds of the depressed subjects were diagnosed with physical health comorbidities in a Scottish country-wide primary care study [5], with the disease cluster of cardio-metabolic conditions and depression being one of the most common multimorbidity patterns throughout clinical settings according to a systematic review [6]. Comorbid depression has been associated with incremental decrements in health [7], prolonged hospital stays and higher chance of rehospitalization [8], lower treatment adherence [9], and increased risk of mortality [10, 11]. Thus, endorsement of the statement “no health without mental health” should readily translate into evidence-based clinical practice guidelines that consider physical and mental health comorbidities [12].

During the past decades, the Collaborative Care Model (CCM) has proven successful in integrating behavioral health services into primary care [13]. Under this model of care, mental health care is provided in a coordinated fashion with the support of a case manager, supervision by a consultant psychiatrist, and the use of patient-reported outcome measures to tailor clinical decision-making to patients’ needs [13]. Studies have consistently reported that the CCM improves depressive symptoms, health-related quality of life, and social functioning of depressed individuals, with no net increase in health care costs [14]. CCM is effective in the treatment of people with depression alone or with comorbid medical conditions [15]; and it might be beneficial for glycemic and blood pressure control in depressed patients with poorly controlled chronic diseases [16]. Additionally, the current literature on CCM has identified some essential components for the implementation of effective integrated disease management programs, such as the inclusion of cognitive-behavioral therapy and problem-solving techniques [17], or motivational interviewing interventions [18], self-help resources delivered through the Internet or computer-based applications [19–21], timely case manager follow-up within the first 4 weeks to increase clinical attention and patient engagement [22], and psychiatric consultation for those patients not achieving improvement after 2 months [22]. However, evidence and experts’ recommendations for effective CCM usage come mainly from developed countries [14], and may not wholly apply to developing nations.

Chile is a developing, high-income Latin American country that has made major advances over the last 30 years in the management of medical and mental health conditions. For instance, Chilean governments have made a strong commitment to promote healthy living and prevent chronic diseases through community-based health programs for people covered by public health insurance [23], and have developed a comprehensive community-based mental health care network, integrating mental health into primary care throughout the country [24]. Moreover, based on the success of a randomized controlled trial to treat depression in low-income women in primary care clinics in Chile’s capital city, Santiago [25], a stepped-care program for depression management, which combined medical and psychosocial interventions, was implemented in 2001 and rapidly scaled-up across primary care facilities in the country [24]. Complementarily, the Chilean Regime of Explicit Health Guarantees, a comprehensive health reform enacted in 2005, mandated guarantees of access, quality, opportunity, and financial coverage by public and private health insurance for priority diseases and conditions [24]. Hypertension, type II diabetes, and depressive disorders were part of the first set of 56 priority diseases, ensuring access to an extensive, evidence-based basket of benefits which are structured according to clinical practice guidelines, informing quality, cost-effective primary care practice.

Despite substantial progress towards universal health access and coverage in Chile [26], especially for depression [24], effective coverage for hypertension and diabetes remains particularly low compared to infectious diseases or maternal and child care [26]. Furthermore, according to the Chilean National Health Survey 2016–2017, there has been a sustained increase in the proportion of people with diabetes [27], while no variation has been observed for the population prevalence of depression [28]. These figures may be attributable to the minimum impact of preventive interventions, as evidenced by the prevalence of cardiovascular risk factors [26], or to important inconsistencies in the provision of treatment, as in the case of depression, where diagnostic inaccuracy and treatment dropout are the main problems in the management of depressed primary care patients [29, 30]. Moreover, while 80% percent of depressed patients at primary care clinics had comorbidity [31], and depressive symptoms are common among hypertensive patients [32], the Chilean clinical practice guidelines for cardio-metabolic and depressive disorders do not consider their common comorbidity in primary care settings. Thus, the country’s epidemiological and health services profile, characterized by a tendency towards ageing, highly prevalent non-communicable diseases, and mental disorders, motivates the urgent search for effective primary care interventions integrating a mental health component in the management of chronic diseases.

## Objectives

### General objective

To compare the effectiveness of a collaborative, computer-assisted, psycho-educational intervention, called “Me cuido y me siento mejor” [I Take Care of Myself and Feel Better], versus enhanced usual care in the treatment of depressed patients with hypertension and/or diabetes in primary care clinics in Santiago, Chile.

### Specific objectives

- To compare the proportion of patients with improvement in depressive symptoms treated with the “Me cuido y me siento mejor” intervention versus enhanced usual care in primary care clinics.
- To compare the proportion of patients recovered from depression treated with the “Me cuido y me siento mejor” intervention versus enhanced usual care in primary care clinics.
- To compare the levels of health-related quality of life and social problem-solving skills in patients treated with the “Me cuido y me siento mejor” intervention versus enhanced usual care in primary care clinics.
- To compare the values of blood pressure and/or glycosylated hemoglobin of patients treated with the “Me cuido y me siento mejor” intervention versus enhanced usual care in primary care clinics.
- To compare treatment acceptability of the “Me cuido y me siento mejor” intervention versus enhanced usual care in primary care clinics.

### Hypotheses

- The proportion of patients with improvement in depressive symptoms will be 23% higher in the “Me cuido y me siento mejor” primary care clinics compared to the enhanced usual care primary care clinics, at 6-month follow-up.
- The proportion of patients recovered from depression would be higher in the “Me cuido y me siento mejor” compared to the enhanced usual care primary care clinics, at 6-month follow-up.
- Patients in the “Me cuido y me siento mejor” primary care clinics would have better social problem-solving skills and health-related quality of life than patients in the enhanced usual care primary care clinics, at 6-month follow-up.
- Patients in the “Me cuido y me siento mejor” primary care clinics would have lower blood pressure and improved glucose regulation than patients in the enhanced usual care primary care clinics, at 6-month follow-up.
- Patients in the “Me cuido y me siento mejor” primary care clinics would report higher treatment acceptability than patients in the enhanced usual care primary care clinics, at 6-month follow-up.

## Methods: participants, interventions, and outcomes

### Study setting

Eight primary care clinics located in two low-to-middle income urban municipalities belonging to the Greater Santiago Metropolitan Area, Chile.

### Eligibility criteria

#### Primary care clinics

*Inclusion criteria:*

- Located in urban municipalities belonging to the Greater Santiago Metropolitan Area, Chile.
- Have implemented a Cardiovascular Health Program and a Depression Program.
- Have higher than the median population of patients enrolled in the Cardiovascular Health Program among all primary care clinics in the Greater Santiago Metropolitan Area.

### Trial design

Multicenter, parallel-group, two-arm, superiority, cluster randomized trial with 1:1 allocation ratio.

#### Trial participants

*Inclusion criteria:*

- Age 18 or older.
- Enrolled in the Cardiovascular Health Program (i.e. currently receiving treatment for diabetes and/or hypertension) at the study primary care clinics.
- Patient Health Questionnaire-9 Item (PHQ-9) score ≥ 15.
- Signed or verbal informed consent.

*Exclusion criteria:*

- Functional illiteracy (i.e., patients unable to read and comprehend written information, such as the study questionnaires or written/verbal informed consent).
- Significant visual and/or auditive impairments (i.e., such as those imposing a serious difficulty to respond the study questionnaires or written/verbal informed consent).
- Pregnancy or breastfeeding.
- Cognitive impairment – ineligible patients would give a negative answer to questions “What year is it?” and “Where are we (place/address)?”.
- In treatment for bipolar and/or psychotic disorder.
- Current psychological treatment for depression.
- High risk of developing alcohol/substance abuse problems, according to an Alcohol, Smoking and Substance Involvement Screening Test (ASSIST) score ≥ 27 [33].

#### Who will take informed consent?

Trained recruiters from the trial will contact potential participants for assessment of the initial eligibility criteria for the study. During the evaluation process, recruiters will explain general aspects of the trial to potential participants and will obtain written (or verbal) informed consent from them.

#### Additional consent provisions for collection and use of participant data and biological specimens

Not applicable.

### Interventions

#### Explanation for the choice of comparators

Currently, in Chile, the standards for the management of depression in primary care have been set in the Clinical Guidelines for the Treatment of Depression, issued by the Chilean Ministry of Health [34]. This document summarizes the best evidence available and is periodically updated by a group of local experts. The target population of the Clinical Guidelines for the Treatment of Depression is people aged 15 years or more. Its aims are to guide practitioners in the active detection and integral diagnosis of the pathology while also offering evidence-based recommendations for timely, efficient, and cost-effective depression treatment [34]. The Clinical Guidelines for the Treatment of Depression incorporates decision-making algorithms in which primary care plays a relevant role, enhancing primary care team’s capacities to manage cases of mild, moderate, and severe depression [34]. Furthermore, the Regime of Explicit Health Care Guarantees, establishes a set of guarantees and rights for people with public or private health insurance [35], offering a comprehensive basket of health benefits according to depression severity. In this context, usual care for depression is the reference comparator in Chile [34]. In the present trial, usual care has been complemented with online access to information about depression, diabetes and hypertension, and healthy lifestyles for the participants.

#### Intervention description

Patients in the primary care clinics assigned to the intervention and to the comparator will have access to the project’s website, which will be populated with information about the project’s aims, the research team, and contact data, along with educational material related to depression, diabetes and hypertension, and healthy lifestyles.

#### Active arm

“Me cuido y me siento mejor” is a multicomponent, collaborative, computer-assisted, psycho-educational intervention for the management of depressive symptoms in primary care patients with diabetes and/or hypertension. Patients in the primary care clinics assigned to the intervention will receive:

- A computer-assisted, psycho-educational intervention, delivered in-person –or remotely if this is not possible– by trained therapists participating in the trial, at the selected primary care clinics. Eight individual sessions will be held in total, each lasting 45 min. Sessions will be held weekly at first (4 sessions), followed by 4 monthly sessions. The intervention manual will ensure standardized implementation of each session. The manual carefully details the aims, activities, necessary materials, and possible conversations with the patient in each session. The therapists participating in the trial will deliver this intervention with the support of a laptop computer to display slides or videos. This intervention will combine education about health issues (depression, diabetes, and hypertension), basic elements of motivational interviewing, and cognitive-behavioral techniques to involve the patients in the management of their own health issues, encourage behavioral activation, improve problem-solving skills, and modify negative thoughts. The first two sessions, based on health education and motivational interviewing, will actively involve the patients in managing their health issues and will establish a link between depression and chronic diseases, specifying their impact on the patients’ quality of life. Also, a personalized care plan will be developed. The patients will receive support materials such as calendars and a blackboard-type care plan. Sessions three and four will address negative thoughts and problem-solving techniques. The fifth session will incorporate elements of behavioral activation, while the sixth will pay closer attention to thinking errors. In all sessions, the patients’ progress in their care plan will be reviewed. The last two sessions (reinforcement sessions) will elaborate on the associations between the topics covered in each session and will feature the closing activities. The therapists will record information about the topics covered in the sessions in the electronic health records of the participating primary care clinics. The therapists will all be psychologists. Their training will consist in a 12-h induction about the basics of the intervention. This training program will include teaching sessions, role playing, and knowledge tests, which will be in charge of the research team. The therapists will participate in a biweekly supervision by a psychologist from the research team to solve logistical problems and issues with treatment integrity.
- Telephone monitoring will be in charge of a social worker participating in the trial. Monitoring calls lasting 5 to 10 min will be made during the first 12 weeks of the intervention, once per week, in order to observe the patients’ clinical evolution, the medical care received, and their use of antidepressants and possible collateral effects, while also encouraging adherence to the medical and psycho-educational treatment. The social worker in charge of telephone monitoring, who has a long experience in managing cases in clinical trials for depression treatment, will have a standardized form for recording information.
- A psychiatrist from the research team will review the social worker’s caseload to formulate treatment recommendations and solve health care problems affecting the patients throughout the study. In addition, the psychiatrist will monthly supervise the study therapists to assist them in the management of the most severe cases and in the collaboration with routine primary care.
- Once per month, a member of the research team will meet with the teams from the participating primary care clinics. These meetings will be aimed at integrating the mental health care provided by the study therapists with the usual medical care for chronic diseases provided in the cardiovascular health program of each primary care clinic, to ensure continuity of care.
- Usual medical care for chronic diseases, according to the Cardiovascular Health Program.

#### Control arm

The patients in the primary care clinics assigned to the comparator will receive the usual treatment for depression and their physical conditions –all the guaranteed interventions for people with depression, hypertension, and/or diabetes in primary care, according to the Clinical Guidelines for the Treatment of Depression– and their associated basket of health benefits included in the Regime of Explicit Health Care Guarantees [34, 35].

In the specific case of depression, in Chile, this pathology is managed using a stepped care model, with more severe cases being addressed with more intense and frequent interventions [34]. The severity of depression is determined with the diagnostic criteria of the International Classification of Diseases, tenth version (ICD-10), which characterizes mild, moderate, and severe levels depending on the number of symptoms [34]. Mild cases are treated with low-intensity interventions such as counseling, support groups, and physical exercise programs; moderate cases are complementarily treated with antidepressants, depending on patients’ response and tolerance; finally, the treatment of severe cases also includes psychotherapy [34]. Depression cases with high suicide risk or psychotic symptoms, or which are refractory to treatment, are referred to psychiatric assessment or to specialized mental health services [34].

#### Criteria for discontinuing or modifying allocated interventions

Primary care team will be informed If patients assigned to the intervention or to the comparator present high suicide risk or a worsening of their depression during the trial.

Interventions will also be interrupted if a patient is hospitalized as a result of a worsening of his/her physical issues.

#### Strategies to improve adherence to interventions

Adherence to trial protocols will be ensured by training and supervising the therapists taking part in the trial. In addition, the therapists and the social worker will be asked to keep a record of each session with the patients, which will be reviewed by a psychiatrist from the research team to ensure adherence to the protocols. Lastly, 10% of the participants will be contacted by a blinded evaluator who will administer an ad-hoc questionnaire designed to assess the therapists’ fidelity to the intervention.

#### Relevant concomitant care permitted or prohibited during the trial

In those primary care clinics assigned to the intervention, usual psychological treatment will be replaced by the psycho-educational intervention.

#### Provisions for post-trial care

The risks associated with the intervention are minimal; therefore, this clinical trial does not entail a compensation for the participants. In addition, health care staff will receive training and materials associated with the intervention in all the participating primary care clinics at the end of the trial follow-up period.

## Outcomes

### Primary outcome

#### Depression improvement

Defined as a 50% reduction in a participant’s Patient Health Questionnaire-9 Item (PHQ-9) score compared to the baseline [36]. The summary measure for each group will be the percentage of participants who respond to their depression treatment at 6-month follow-up. Even though the primary outcome is taken at 6-month follow-up, this measure will also be reported at 3- and 12-month follow-up.

### Secondary outcomes

#### Depression recovery

Defined as a change in a patient’s depressive status from an initial score above the cutoff (PHQ-9 ≥ 10) and a follow-up score below this reference value (PHQ-9 < 10). The summary measure for each group will be the percentage of participants who respond to their depression treatment at 3-, 6-, and 12-month follow-up.

#### Health-related quality of life

Defined as a patient’s score on the scales and main components of the Short-Form 12 Health Survey (SF-12) [37]. The summary measure for each group will be the mean score of the participants at 3-, +-, and 12-month follow-up.

#### Social solving problem skills

Defined as a patient’s score on the Positive Problem Orientation and Rational Problem Solving Style subscales of the Social Problem-Solving Inventory – Revised Short Form (SPSI-R:S) [38]. The summary measure for each group will be the mean score of the participants at 3-, 6-, and 12-month follow-up.

#### Blood pressure and glycosylated hemoglobin

Defined as the achievement of therapeutic goals in the normalization of blood pressure and glycemic control, according to the national standards established in the Clinical Guidelines of Primary or Essential Arterial Hypertension in people aged 15 or older and in the Clinical Guidelines for Type II Diabetes Mellitus, respectively. With respect to arterial pressure, the therapeutic goal is to attain a value lower than 140/90 mmHg in non-diabetic patients and lower than 130/80 mmHg in patients with very high cardiovascular risk, diabetes, and/or proteinuric nephropathy. Maintaining levels of glycosylated hemoglobin below 7% is regarded as a therapeutic goal in type II diabetes mellitus. The summary measure for each group will be the percentage of participants who achieve these blood pressure and glycemic control goals at 3-, 6-, and 12-month follow-up.

#### Acceptability of depression treatment

Defined as the score on an ad-hoc instrument for measuring the acceptability of depression treatment. The summary measure for each group will be the mean score of the participants at 3-, 6-, and 12-month follow-up.

### Other measures

Complementarily, we will evaluate the participants’ utilization of health care services and treatment adherence at 3-, 6-, and 12-month follow-up.

### Participant timeline

**Fig. 1:**
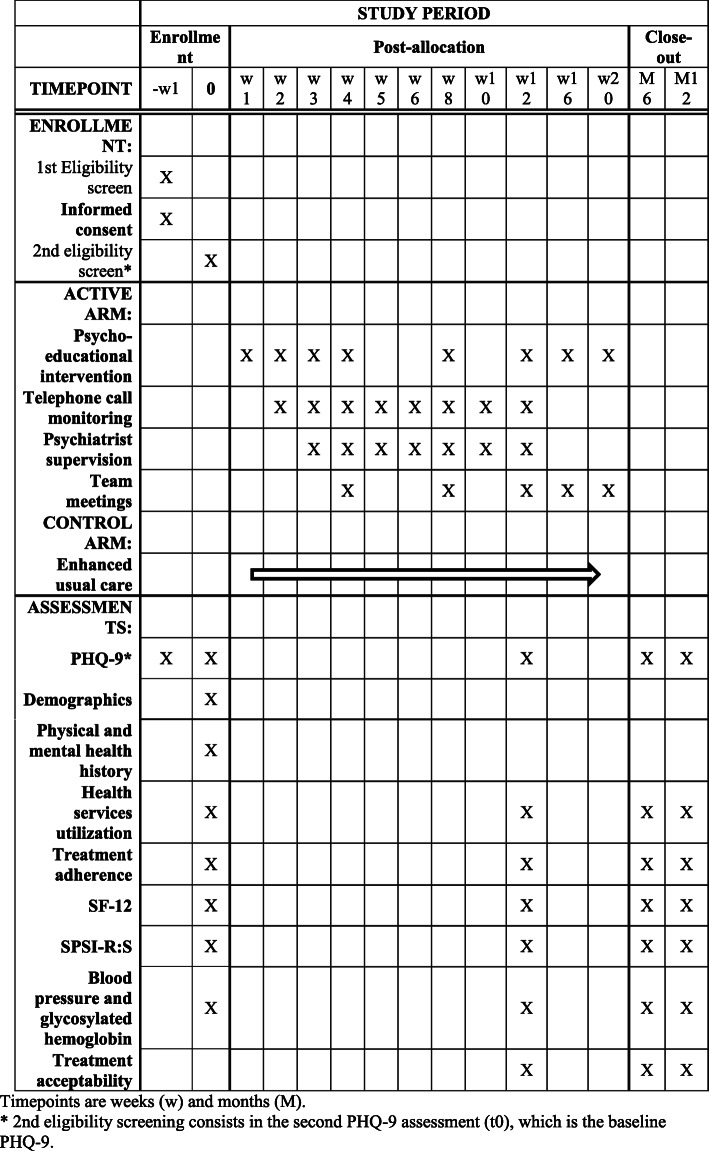
Participant timeline

The participants’ timeline is shown in Fig. 1.

### Sample size

The meta-analysis conducted by Huang et al. [39], about the effectiveness of collaborative care in diabetic and depressed patients treated in primary health care showed that 27% of patients in the usual group responded to depression treatment. Considering a two-tailed alpha of 0.05, a statistical power of 80%, 4 clusters per treatment group, 38 participants per cluster, and assuming an intraclass correlation coefficient (ICC) of 0.02 –a more conservative ICC than that reported by Adams et al.– [40] with a design effect of 1.74, we calculated a minimum detectable difference of 23% between the treatment groups, with the expectation that 50% of the members of the active group will respond to depression treatment. Anticipating 15% attrition at follow-up, this study will need to recruit 45 participants in each of the 8 clusters (360 cases in total).

### Recruitment

A trained recruiter will be allocated to each of the primary care clinics taking part in the trial. Recruiters will contact potential participants in-person – at the primary care clinics’ waiting rooms – or remotely (e.g., via telephone call and based on lists of registered cardiovascular patients at the primary care clinics), if in-person contacts are not possible. Recruiters will inform patients of the study, evaluating the initial eligibility criteria and administering the informed consent. After securing the patients’ written informed – or verbal, if in-person contact is not possible –, and if they meet the initial eligibility criteria (e.g. PHQ-9 ≥ 15), potential participants will be contacted again by the recruiters for an in-person (or remote – e.g., telephone call) assessment 1 week later. During this period, recruiters will send reminders to potential participants using the available means of contact. In the reassessment, those with a PHQ-9 ≥ 15 will be recruited for the trial and will be invited to continue by answering the baseline evaluation.

To ensure a sufficient recruitment flow, recruiters will work every day of the week in the morning, when most people attend health care centers. These activities will be supervised by a person in charge of recruitment, who will be in close contact with members of the research team in charge of fieldwork. To facilitate the work of the recruiters, signs, leaflets, and banners with basic information about the study will be on display, inviting potential participants to contact the recruiters. Each recruiter will remain in the allocated primary care clinic until he/she has recruited 45 participants. Additionally, if in-person recruitment is not possible, the lists of registered cardiovascular patients at the primary care clinics will be obtained for the remote contact of potential participants.

### Assignment of interventions: allocation

#### Sequence generation

In each municipality, the four participating primary care clinics will be allocated to the intervention or the comparator with a 1:1 ratio based on random computer-generated numbers, using simple random sampling. This sampling method was used instead of others (e.g. stratified) because health care centers in each municipality tend to operate homogeneously and serve population with similar characteristics.

#### Concealment mechanism

Allocation will be performed at the clinic level, not the participant level. To minimize the possibility of selection bias at the cluster level, the health care clinics will be identified and recruited prior to randomization. Cluster allocation will be concealed from administrative staff of the primary care clinics who could come into contact with the recruiters. Likewise, cluster allocation will be concealed from the recruiters [41].

#### Implementation

A member of the research team who is not involved in the recruitment process in primary care clinics will be in charge of the generation of the random sequence and the allocation of the clusters to the interventions. Cluster allocation will be kept concealed from the recruiters; to do this, the process will be hidden from administrative staff of the primary care clinics who could come into contact with the recruiters, who will be expressly instructed not to contact health care personnel at the participating centers.

### Assignment of interventions: blinding

#### Who will be blinded

Due to the nature of the interventions, it will not be possible to blind the providers or the patients who participate in the trial. Outcome assessments will be conducted by an evaluator blinded to the allocation of the interventions. These evaluators will be trained to administer the battery of evaluation instruments, but will not be given any details about the procedures involved in the study.

#### Procedure for unblinding if needed

Not applicable.

### Data collection and management

#### Plans for assessment and collection of outcomes

Technical staff in charge of the assessments and data collection will be selected considering their prior experience in survey administration. In addition, they will participate in a training session focused on the administration of the battery of instruments of the trial and will receive permanent supervision from the person in charge of recruitment and/or research team members. Specifically, baseline evaluations will be conducted by the recruiters, while follow-up evaluations (3, 6, and 12 months) will be carried out by the outcome evaluators. The baseline and outcomes assessments will be conducted in-person – or remotely, if this is not possible (e.g., telephone calls).

The outcomes section lists the measurement instruments for each follow-up assessment: PHQ-9, SF-12, SPSI-R:S, an ad-hoc instrument for assessing the acceptability of depression treatment, and a questionnaire about the utilization of health care services and treatment adherence. These instruments will be described below. Blood pressure and glycosylated hemoglobin data will be retrospectively collected from the clinical records of each participant by outcome evaluators blinded to the allocation of primary care clinics.

The PHQ-9 is one of the world’s most widely used measures for self-reporting depressive symptoms. It has been validated for use with primary care populations, which will make it possible to study major depression as an estimate of the intensity of depressive symptoms (i.e. severity of the condition) [36]. The PHQ-9 comprises nine questions based on the diagnostic criteria for depressive episodes described in the Diagnostic and Statistical Manual of Mental Disorders, fourth edition (DSM-IV); each of these questions has four answer choices, which make it possible to evaluate the intensity of each symptom [36]. The scale ranges from 0 to 27 points, with the following gravity classification: no depression (0 to 4 points), mild depression (5 to 9 points), moderate depression (10 to 14 points), moderate-severe depression (15 to 19 points), and severe depression (20 or more points). In the original validation study for its use in primary care, a cutoff score of 10 points or more had a sensitivity and specificity of 88% for major depression [36]. The PHQ-9 has been validated for use with primary care populations in Chile [42].

Nowadays, the multiple versions of SF questionnaires (i.e. 8, 12, and 36) are the reference standard for assessing health and well-being from the patient’s perspective, having been validated for use with a variety of populations worldwide. Specifically, the SF-12 –a reduced 12-item version of the SF-36– is easier to understand and administer and is less taxing for patients than the original version [37]. The SF-12 comprises eight scales (physical functioning, role-physical, bodily pain, general health, vitality, role-emotional, social functioning, and mental health) and two principal components (physical component summary and mental component summary), with items that have a 4-week recall period and scores ranging from 0 (worst health possible) to 100 (best health possible) [37]. The SF-12 has been validated for use with beneficiaries of public and private health care in Chile [43].

The SPSI-R:S is a self-report instrument that assesses individual social problem resolution skills based on the five-component Model of Social Problem-Solving developed by D’Zurilla and Nezu [38]. This questionnaire has been widely used around the world, is easy to administer, and has good psychometric properties. The SPSI-R:S comprises 25 Likert-type items with five answer choices ranging from 0 (not at all true) to 4 (extremely true), and has five subscales: Positive Problem Orientation, Negative Problem Orientation, Rational Problem Solving Style, Impulsivity/Carelessness Style, and Avoidance Style [38].

The ad-hoc instrument for evaluating the acceptability of the interventions is a self-report questionnaire that consists in 12 Likert-type items with four answer choices ranging from 1 (strongly agree) to 4 (strongly disagree). The construction of this instrument was based on the theoretical framework of acceptability, proposed by Sekhon et al. [44], which characterizes seven dimensions for this concept: ethicality, affective attitude, burden, opportunity costs, perceived effectiveness, self-efficacy, and intervention coherence. For their interpretation, the participants’ scores were transformed into percentages (0 to 100%), with higher values representing more intervention acceptability.

The questionnaire about the utilization of health care services, adapted from the National Socioeconomic Characterization Survey [45], has a 3-month recall period and records physician consultations or treatments, primary care and/or hospital emergencies, specialist medical treatment or mental health treatment, and hospitalizations, noting the total number of sessions (total days for hospitalizations) and the payment made for each session. In addition, two self-report questionnaires will be administered to evaluate treatment adherence: and ad-hoc questionnaire and the Batalla Test [46]. The ad-hoc questionnaire will comprise a series of dichotomous questions about medication adherence. The Batalla Test, on the other hand, evaluates the patient’s knowledge about his/her disease with three questions, assuming that more patient knowledge reflects more adherence.

#### Plans to promote participant retention and complete follow-up

This clinical trial will provide no incentives to increase participant retention. To reduce the burden on participants, they will be given the choice to attend in-person evaluations (at home or in a health care center) and/or receive telephone calls. The technical staff in charge of the evaluation and the data collection, along with the clinicians that deliver the interventions, will be reminded to keep an up-to-date record of the participants’ contact information in order to prevent data loss during the follow-up stage. Participants who decide to withdraw from the study will no longer complete follow-up evaluations. An informed consent revocation form will be provided for these cases.

#### Data management

The data collected from each participant will be directly stored in electronic forms by the recruiters or the outcome evaluators during the trial. After completion, these electronic forms will be submitted online, through a secure platform, to be automatically downloaded to a unique and encrypted database. No copies of the data will be kept on the electronic devices used in the study (e.g. smartphones, tablets, personal computers). Data will be stored in a centralized database by the researchers in charge of administering trial data. Data verification procedures will be conducted weekly to identify missing or mistaken values.

#### Confidentiality

Participant’s sensitive data and identification will be replaced with a set of unrelated characters (ID code). This will make it possible to work with coded and anonymized data, thus safeguarding the participants’ confidentiality. As detailed in the “Data management” section, data will be encrypted and securely kept, with access being supervised and centralized by the researchers in charge of administering trial data, who will access them using passwords.

#### Plans for collection, laboratory evaluation and storage of biological specimens for genetic or molecular analysis in this trial/future use

Not applicable.

### Statistical methods

#### Statistical methods for primary and secondary outcomes

Results will be reported according to the Consolidated Standards for Reporting Trials (CONSORT), specifically its extensions for cluster randomized clinical trials and clinical trials of non-pharmacological interventions [47, 48]. We will check the balance between the baseline characteristics of the intervention groups. As a general rule, for dichotomous or categorical variables, we will present the percentage and frequency of each category of interest (e.g. response vs. no response to treatment) for each intervention group, along with the odds ratio (OR) as a measure of the effect, a 95% confidence interval (95% CI), and *p* value. For continuous variables, we will present the means and standard deviations for each intervention group, along with the mean difference between the groups, 95% CI, and *p* value. For the analysis of dichotomous outcomes (including the main outcome), we will fit logistic random effects regression models to reflect the grouped nature of the data and will report OR, with their respective 95% CI, as effect size measures. The same strategy will be used for continuous outcomes (i.e. difference in means between the groups) with linear random effects regression models and Cohen’s *d* (i.e. standardized mean difference), with their respective 95% CI, as effect size measure. These analyses will be assisted using Stata 14.0 [49].

#### Interim analyses

No interim analyses are planned.

#### Methods for additional analyses (e.g. subgroup analyses)

Regarding the evaluation of the effects of the intervention (i.e. analysis of the main and secondary outcomes), raw and adjusted effect estimates will be presented. The adjustment will involve sociodemographic variables with a well-known diagnostic value (i.e. sex and age), complemented with covariables to reflect the potential imbalance between the intervention groups.

Additional analyses will consider the fit of repeated measures mixed effects models to determine whether the effect of the intervention differs over time, taking into account the correlation between the measures of a single participant, as well as the study design. The potential moderating effects of sex and age will be considered in these analyses.

#### Methods in analysis to handle protocol non-adherence and any statistical methods to handle missing data

All analyses will be performed according to the principle of intention-to-treat (ITT), which means that all participants are included in the analysis according to the group that were randomly allocated, regardless of the treatment received. Complementarily, per-protocol analyses will be conducted. This group of analyses will include patients who have received at least 85% of the total number of interventions scheduled in the protocol.

In clinical trials, missing data are the rule, not an exception. Therefore, the specialized literature reports the superiority of modern imputation methods (i.e. multiple imputation [MI]) compared to other analysis methods (e.g. complete-case analysis [CCA]) [50]. In the present trial, we will estimate the mechanism of missingness. If found to be pertinent, we will apply the MI method following the procedures described by Enders, Keller, and Levy [51], and Bartlett, Seaman, White, and Carpenter [52], in their extension for the management of grouped data in Blimp 1.1 [51, 53]. These procedures make it possible to manage random slopes, categorical and nominal variables, the disaggregation of inter- and intra-subject effects, and incomplete level 2 variables [51]. The data analysis and combination of the sets of imputed data will be conducted with Stata 14 [49], adjusting the regression coefficients and standard errors according to the combination rules developed by Rubin [54].

#### Plans to give access to the full protocol, participant level-data and statistical code

Access to the full protocol, participant level-data, and statistical code upon reasonable request to the corresponding author.

### Oversight and monitoring

#### Composition of the coordinating Centre and trial steering committee

GR will coordinate and lead the research project, supervising the research team and the technical support personnel. She will supervise the data analysis and the dissemination of results. She will be in charge of organizing international cooperation efforts.

PM will be in charge of liaisons with the participating institutions and primary care clinics, intervention design, fieldwork coordination and support, data administration and analysis, and result dissemination.

VG will contribute to intervention design, database generation, data analysis, and result dissemination.

SC will be involved in the design of the intervention, the training and supervision of the study therapists, while also contributing to data analysis and result dissemination.

PH will help design the intervention, will be in charge of training and supervising the therapists, and will administer the evaluations of intervention acceptability and fidelity.

PV will supervise the trial from a methodological point of view, will be in charge of randomization and instrument selection, will supervise the generation of databases and their analysis, and will help with result dissemination.

VG will contribute to the coordination of the intervention.

RA will provide methodological assistance during the trial.

#### Composition of the data monitoring committee, its role and reporting structure

As this trial entails minimal risks to the patients, no data monitoring committee will be established.

### Adverse event reporting and harms

The risks associated with the intervention are minimal; therefore, no adverse events are expected. The follow-up evaluations of all the participants cover suicide risk (i.e. question 9 of the PHQ-9) and depressive symptoms. If high suicide risk is observed or if a patient’s depression significantly worsens during the trial, we will consider the processes described in the section “Criteria for modifying or discontinuing allocated interventions”. This information will be duly notified to the affected participant, the treating physicians at the primary care clinics, and the Scientific Ethics Committee of the Clinical Hospital of the Universidad de Chile.

#### Frequency and plans for auditing trial conduct

No audits have been scheduled to be implemented during the trial.

#### Plans for communicating important protocol amendments to relevant parties (e.g. trial participants, ethical committees)

Any amendments to the trial protocol which may affect the safety of the participants must be communicated to and approved by the Scientific Ethics Committee of the Clinical Hospital of the Universidad de Chile.

#### Dissemination plans

The results of the clinical trial will be communicated to all interested parties regardless of the magnitude or direction of the effect found. Patients will be informed of the availability of the results of the clinical trial at the website of the research project, using accessible language and safeguarding their confidentiality. In addition, meetings will be held with the health care teams of the participating primary care clinics in order to return the information obtained to them, highlighting the clinical implications of the results of the trial. Furthermore, the primary and secondary outcomes of this clinical trial will be made available to the scientific community in a peer-reviewed journal devoted to the topic of interest (i.e. mental health).

## Discussion

The design of the collaborative, computer-assisted, psycho-educational intervention *“Me cuido y me siento mejor”* considered the findings of a pre-post pilot study, with no control group, whose (still unpublished) results are promising. In brief, the pilot version of the intervention displayed high adherence and acceptability, demonstrating its potential effectiveness for reducing depressive symptoms and the utilization of health care services, while also increasing adherence to diabetes and/or hypertension treatment. This suggests that *“Me cuido y me siento mejor”* may be effective in treating depression in patients with chronic diseases in primary care; also, importantly, it may facilitate access to the behavioral changes that patients with diabetes and/or high blood pressure need. If these behavioral changes could be sustained over time, patients who receive this intervention would be expected to display improvements in terms of physical health indicators (such as arterial pressure and/or glycosylated hemoglobin). In the present trial, this will be tested through a follow-up period lasting up to 12 months. Complementarily, the piloting of this trial made it possible to adjust the evaluation procedures (e.g. by shortening the result evaluation forms to reduce the participants’ burden) and the design of the intervention. Regarding this last point, and considering the patients’ opinion, the frequency and number of sessions were increased in order to incorporate contents about behavioral activation and reinforce the use of problem-solving techniques. Lastly, it should be noted that this is one of the first initiatives focused on the collaborative management of depression and chronic diseases in primary care in Latin America.

## Data Availability

The datasets used and/or analysed during the current study are available from the corresponding author on reasonable request.

## Abbreviations

WHO: World Health Organization
CCM: Collaborative Care Model
PHQ-9: Patient Health Questionnaire-9 Item
ASSIST: Alcohol, Smoking and Substance Involvement Screening Test
ICD-10: International Classification of Diseases
SF-12: Short-Form 12 Health Survey
SPSI-R:S: Social Problem-Solving Inventory – Revised Short Form
ICC: Intraclass Correlation Coefficient
DSM-IV: Diagnostic and Statistical Manual of Mental Disorders, fourth edition
CONSORT: Consolidated Standards for Reporting Trials
OR: Odds Ratio
95% CI: 95% confidence interval
MI: Multiple Imputation
CCA: Complete-Case Analysis
